# SKA3 Up-regulation Promotes Lung Adenocarcinoma Growth and is a Predictor of Poor Prognosis

**DOI:** 10.1515/biol-2019-0044

**Published:** 2019-07-30

**Authors:** Rong-Li Sun, Feng-Juan Liu, Xiao Wu, Li-Sheng Wang, Peng-Fei Wang, Chun-Ling Zhang

**Affiliations:** 1 Department of Respiratory, The Affiliated Central Hospital of Qingdao University, No.127 Siliu South Road, Qingdao, Shandong 266042, P.R. China

**Keywords:** SKA3, lung adenocarcinoma, prognosis, migration

## Abstract

**Objective:**

The objective of this research is to investigate the expression and function of SKA3 in lung adenocarcinoma.

**Methods:**

The mRNA expression level of SKA3 in lung adenocarcinoma and its association with clinic-pathological factors were analyzed using data obtained from the TCGA database. Small interfering RNA (siRNA) for SKA3 (si-SKA3) was used to down-regulate SKA3 in A549 cells. pcDNA3.1- SKA3 was used to overexpress SKA3 in A549 cells. The proliferation ability of A549 cells was determined via MTT assay and colony formation assay. A wound healing assay was performed to examine the migration ability of A549 cells. The protein expression of p-MEK, MEK, p-ERK and ERK were determined by western blot.

**Results:**

We found that SKA3 is up-regulated in lung adenocarcinoma compared to the normal lung tissues. Kaplan-Meier analysis showed that high SKA3 expression is markedly associated with poor prognosis in lung adenocarcinoma patients. SKA3 expression is significantly correlated with age, gender, pathologic-stage, pathologic-N and pathologic-M. Moreover, depleting SKA3 obviously inhibited A549 cell proliferation and migration *in vitro*, while overexpression of SKA3 notably increased A549 cell proliferation and migration. Western blot analysis showed that the protein expression ratio of p-MEK/MEK and p-ERK/ERK decreased noticeably after depleting SKA3.

**Conclusion:**

SKA3 expression was enhanced and associated with poor prognosis in lung adenocarcinoma patients, and it might play a facilitating role in cell growth and motility by regulating the MAPK signaling pathway.

## 1 Introduction

Non-small cell lung cancer (NSCLC) makes up 80% of lung cancers and is becoming one of the main causes of cancer death around the world [1]. Lung adenocarcinoma is the most common histological subtype of NSCLC [2] , which tends to form haematogenous metastasis and then causes patient death [1, 3]. To date, lung adenocarcinoma patients have often showed poor prognoses since only limited access has been available for early detection and effective therapy [4, 5]. Therefore, a deeper understanding of the molecular mechanisms of lung adenocarcinoma is essential for us to develop more effective predictors and therapeutic strategies.

Unbalanced chromosome segregation in mitosis often leads to the formation of aneuploid progeny [6]. Aneuploidy is known as one of the universal features of tumors and is a possible cause of tumorigenesis [6]. Spindle And Kinetochore Associated Complex Subunit 3 (SKA3, also called C13orf3) has been previously reported as a binding partner of the Ska1/Ska2 complex, as well as a novel kinetochore protein [7]. Researchers have identified that SKA3 depletion significantly delays anaphase transition in HeLa S3 cells and indicated that the Ska complex played an essential role in microtubules-kinetochores (MT–KT) attachment and chromosome congression [8]. Similarly, Daum et al. found that HeLa cells with reduced SKA3 expression were arrested at metaphase with aligned chromosomes which couldn’t enter anaphase [7]. Recently, Chuang et al. reported that knockdown of SKA3 significantly inhibited colorectal cancer cell growth and motility, and lead to a G2/M arrest [9]. However, the expression and role of SKA3 in lung adenocarcinoma needs to be further investigated.

In the present research, we analyzed the expression level of **S**KA3 in lung adenocarcinoma tissues, and the association between SKA3 expression and the clinic-pathological features of lung adenocarcinoma patients. Moreover, we also explored the role of SKA3 in lung adenocarcinoma cell growth and migration.

## 2 Methods

### 2.1 Expression and clinical data collection

The expression data of 59 adjacent normal samples and 535 lung adenocarcinoma samples were obtained from TCGA database (https://cancergenome.nih.gov/) and used to analyze SKA3 mRNA expression. Among these 535 lung adenocarcinoma samples, 483 cases had complete clinical data. These clinical data were downloaded and used to analyze the relationship between SKA3 expression and prognosis. The 483 samples were divided into high (N=242) and low expression (N=241) groups according to the median of SKA3 mRNA expression. In addition, we also analyzed the expression of SKA3 in lung adenocarcinomas and normal lungs based on 3 datasets obtained from ONCOMINE database. The 3 datasets were: Hou Lung Statistics (including 65 normal lung samples and 45 lung adenocarcinoma samples), Selamat Lung Statistics (including 58 normal lung samples and 65 lung adenocarcinoma samples), and Okayama Lung Statistics (including 20 normal lung samples and 226 lung adenocarcinoma samples).

### 2.2 Cell lines

Human lung adenocarcinoma cell lines A549 and NCI-H460, and human normal lung epithelial EBAS-2B cell lines were bought from the Cell Bank of the Chinese Academy of Sciences (Shanghai, China). All the cells were grown in Roswell Park Memorial Institute (RPMI)-1640 medium (Corning: Manassas, VA) supplemented with fetal bovine serum (10% FBS), Penicillin (100 U/ml), and streptomycin (100 mg/ml) at 37℃ with 5% CO_2_.

### 2.3 Cell transfection

Small interfering RNAs (siRNAs) for SKA3 (Si-SKA3-1 (5’- GCGACUUUGAAGAUUAUCC-3’) and si-SKA3-2 (5’-GUUCAGACUCUAAAGGAUG-3’)) were synthesized by GENEWIZ, Inc. (Beijing, China) and used to down-regulate SKA3 expression in A549 cells. Scrambled si-RNA ( si-con, 5’- UAUUAGAAGUUCUAGCGCC-3’) was used as the negative control. pcDNA3.1-SKA3 plasmid was constructed to overexpress SKA3. pcDNA3.1 vector was used as negative control. All the transfections were executed with Lipofectamine 2000 (Invitrogen, Carlsbad, CA) following the manufacturer’s instructions.

### 2.4 RNA extraction and real-time quantitative PCR

RT-qPCR was used to determine the mRNA expression level of SKA3 in A549, NCI-H460 and EBAS-2B cells. Total RNA was isolated using RNAiso Plus (TaKaRa Biotechnology, Dalian, China) following the manufacturer’s instructions. HiFiScript cDNA Synthesis Kit (CwBio, Beijing, China) was used to form cDNA. Then we used Applied Biosystems 7300 Sequence Detection System (Applied Biosystems) to perform reverse transcription polymerase chain reaction (RT-PCR) and determine the mRNA expression level of SKA3. The primers used were shown in table 1. GAPDH was used as the endogenous reference gene. The mRNA expression level of SKA3 was calculated by a 2-^ΔΔCt^ method.

**Table 1:** The primers used for qPCR.

Primers	Sequence
SKA3 forward	5’- GGTGTTCTGGGAAGATGGCA-3’
SKA3 reverse	5’-TCTGGAAGGAGCTGAGGACA-3’
GAPDH forward	5’-GGAGCGAGATCCCTCCAAAAT -3’
GAPDH reverse	5’–GGCTGTTGTCATACTTCTCATGG-3’

### 2.5 Cell proliferation assay

MTT assay was implemented to examine the proliferation of A 549 cells. After 48 hrs transfection, A549 cells were seeded into 96-well plates and cultured at 37 ^⍜^C with 5% CO_2_. After being cultured for 24 h, 20 μl of MTT solution (5 mg/ml) was added to each well, and the supernatant was discarded after incubating at 37° C for 4 h. Then 200 μl of DMSO solution was added to each well and incubated on a shaker for 10 min. After the purple colored precipitates of formazan were fully dissolved, the OD value at 490 nm was detected using a microplate reader.

Colony formation assay was performed to further test the viability of A549 cells. Approximately 500 cells were seeded into a 60 mm dish and cultured at 37°C. The medium was changed every 3 days. After being cultured for 14 days, we fixed the cells with 4% paraformaldehyde and stained them with 0.1% crystal violet dye. Finally, the visible colonies were counted. All these experiments were performed in quintuplicate.

### 2.6 Wound healing assay

We then performed a wound healing assay to investigate the effects of SKA3 on cell migration. A horizontal wound was made on the cell monolayer using a micropipette tip. The scratched cells were then removed by PBS washing, followed by plate incubation at 37° C with 5% CO_2_. The widths of the wounds were photographed and measured after 0 h and 24 h scratching. The relative migration distances of A 549 cells in si-SKA3 group were compared to the si-con group.

### 2.7 Western blot analysis

Total protein of A549 cells from different groups were isolated using RIPA lysates (Beyotime, Jiangsu, China). The proteins were separated by 10% SDS-PAGE and electro-transferred to a PVDF membrane (Millipore, Bedford, MA). Afterwards, the proteins on the membrane were blocked by 5% skim milk for 1 h at room temperature followed by incubation with the primary antibodies at 4 ^⍜^C for 16 h. After being washed by TBST for 3 times ×5 min, the membranes were incubated with secondary antibodies (1/1000, Cell Signaling Technology) for 1 h. GAPDH was used as an intrinsic quality control. Finally, the bands were detected using an Enhanced chemi-luminescence (ECL) plus detection kit (Thermo Fisher Scientifc, Inc.). Quantity one software was used to quantify the density of the bands.

### 2.8 Statistical analysis

SPSS 15.0 software was used to carry out the statistical analysis. Student’s t-test, χ2 test and one-way ANOVA analysis were used to evaluate the significance of differences between groups as appropriate. We performed Kaplan-Meier analysis and log-rank test to plot the survival curves and compare the difference between groups. It was considered to be statistically significant when p<0.05.

## 3 Results

### 3.1 SKA3 expression is significantly enhanced in lung adenocarcinoma tissues and lung adenocarcinoma cells

**Figure 1:**
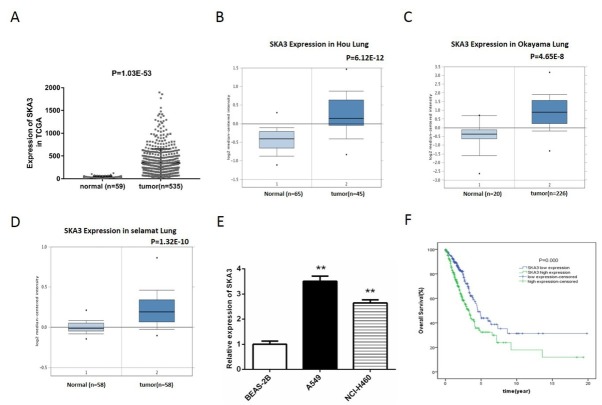
SKA3 expression is up-regulated in lung adenocarcinoma tissues and associated with lower overall survival. (A) SKA3 mRNA expression analysis in lung adenocarcinoma tissues based on the data obtained from TCGA database. (B-D) SKA3 mRNA expression analysis in lung adenocarcinoma tissues based on the data obtained from ONCOMINE database (B: SKA3 Expression in Hou Lung; C: SKA3 Expression in Okayama Lung; D: SKA3 Expression in Selamat Lung). (E) SKA3 mRNA expression in lung adenocarcinoma cells and normal lung cells determined by qPCR. (F) The association between SKA3 expression and overall survival time was analyzed by Kaplan-Meier method based on the TCGA database. The samples were divided into high (N=242) and low (N=241) expression groups based on the median of SKA3 expression.

The expression of SKA3 in lung adenocarcinoma tissues (N=535) and normal tissues (N=59) was analyzed based on the data get from TCGA database. The results showed that SKA3 mRNA expression was enhanced substantially in tumor tissues compared with normal tissues (p=1.03E-53, Figure 1A). Similar results were obtained based on the data from ONCOMINE database. The expression of SKA3 is notably higher in the lung adenocarcinoma samples than in the normal lung samples (p<0.0001, Figure 1B-D). The results of qPCR revealed that the mRNA expression levels of SKA3 in lung adenocarcinoma cell lines A549 and NCI-H460 were significantly higher than in normal lung epithelial EBAS-2B cells (p<0.01, Figure 1E).

### 3.2 Correlations between SKA3 expression and clinic-pathological factors

A total of 483 lung adenocarcinoma cases with complete clinical data were obtained from TCGA database and divided into a high expression group (N=242) and low expression group (N=241) according to the median of SKA3 expression. As shown in table 2, chi-square test analysis demonstrated that SKA3 expression was significantly associated with age (p=0.035), gender (p≤0.001), pathologic-stage (p≤0.001), pathologic-N (p≤0.001) and pathologic-M (p=0.046). No significant correlation was observed between SKA3 expression and pathologic-T (p=0.284).

**Table 2:** The correlation between SKA3 expression and the clinic-pathological characteristics of 483 lung adenocarcinoma patients. *p<0.05

Characteristics	Expression of SKA3		P value
	Low	High	
**Age**			0.035*
<60	56	77	
≥60	185	165	
**Gender**			≤0.001*
female	153	109	
male	88	133	
**Pathologic-Stage**			≤0.001*
I+II	208	175	
III+IV	33	67	
**Pathologic-T**			0.284
T1+T2	214	207	
T3+T4	27	35	
**Pathologic-N**			≤0.001*
N0	181	140	
N1	60	102	
**Pathologic**-M			0.046*
M0	235	227	
M1	6	15	

Afterwards, Kaplan-Meier analysis was implemented to investigate the prognosis value of SKA3 in lung adenocarcinoma. The results indicated that high SKA3 mRNA expression was significantly associated with a lower overall survival rate (p≤0.001, Figure 1F).

### 3.3 SKA3 regulates A549 cell proliferation and migration in vitro

To explore the role of SKA3 in lung adenocarcinoma cell growth, the A549 cells were transfected with SKA3 siRNA to deplete SKA3 expression. As shown in Figure

**Figure 2:**
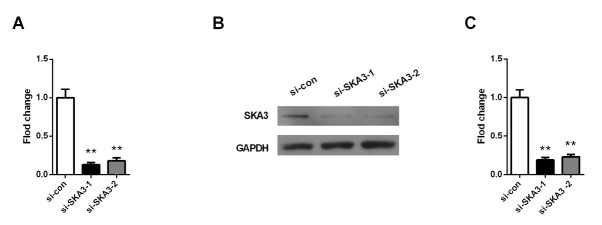
Depleting SKA3 in lung adenocarcinoma A549 cells using siRNA. (A) The mRNA expression level of SKA3 in A549 cells analyzed by qPCR after transfection with siRNA. (B) Western blot was performed to determine the protein expression level of SKA3 in A549 cells after transfection with siRNA. (C) Quantification of SKA3 protein expression using quantity one software. Columns, mean (n=6); bars, SD. **P<0.01, vs. SKA3 expression in A549 cells transfected with scramble siRNA (si-con).

**Figure 3:**
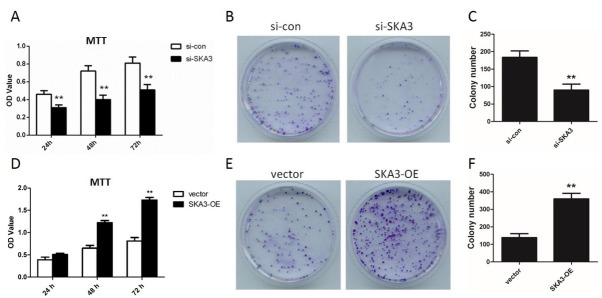
SKA3 promotes A549 cell proliferation. (A) Depleting SKA3 inhibits the proliferation of A549 cells. (B-C) Depleting SKA3 reduced the number of formatted colonies. (D) Overexpression of SKA3 promoted the proliferation of A549 cells. (E-F) Overexpression of SKA3 increased the number of formatted colonies. **p<0.01, vs. si-con group or vector group SKA3-OE: A549 cells transfected with pcDNA3.1-SKA3.

**Figure 4:**
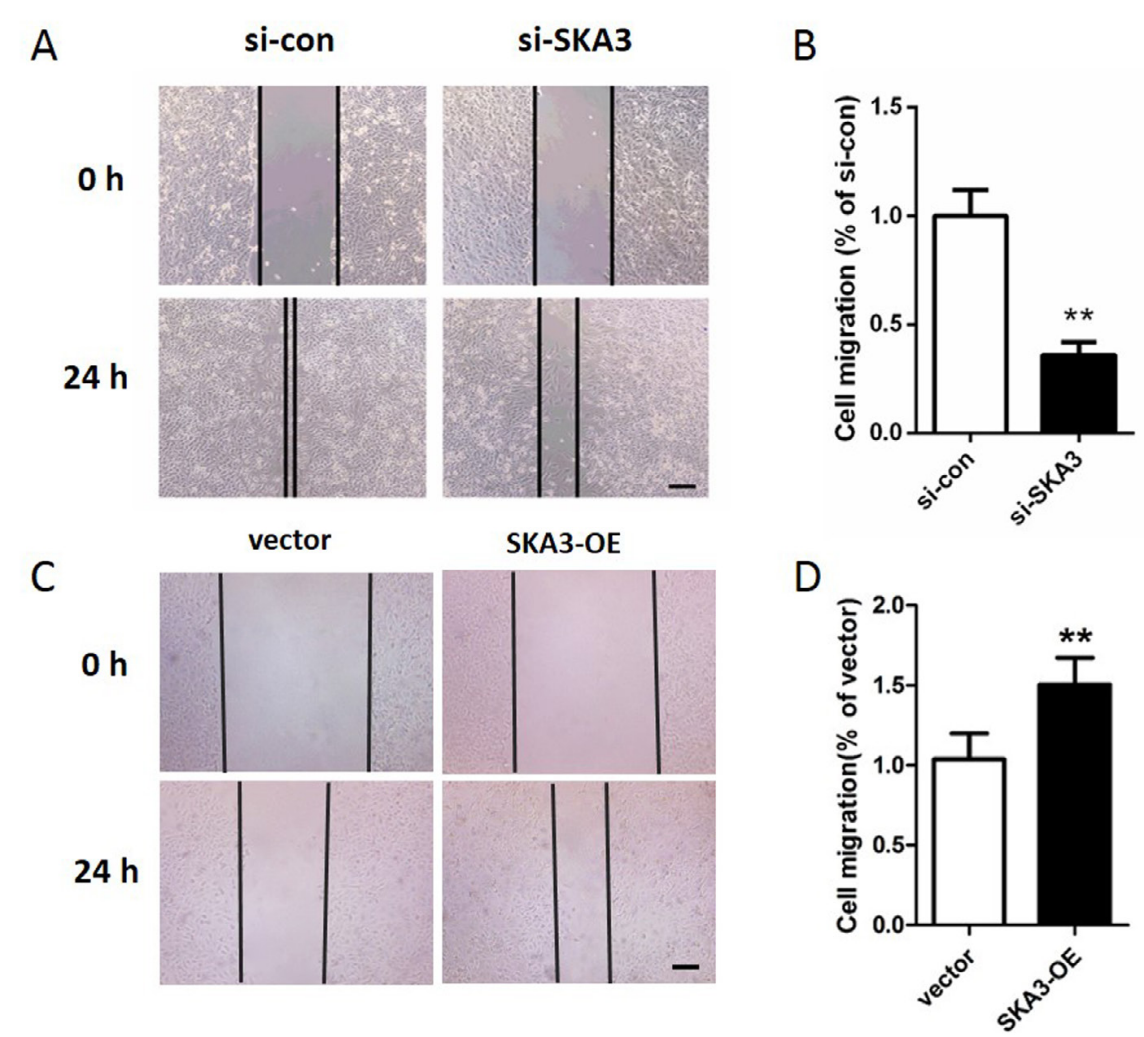
SKA3 promotes A549 cell migration. (A) The effect of silencing SKA3 on cell migration was determined by wound healing assays. (B) Quantification of the migration distance of A549 cells transfected with si-con or si-SKA3. (C) The effect of overexpress SKA3 on cell migration was determined by wound healing assays. (D) Quantification of the migration distance of A549 cells transfected with vector or pcDNA3.1-SKA3. **p<0.01, vs. si-con group or vector group. Bars=200 μm. SKA3-OE: A549 cells transfected with pcDNA3.1-SKA3.

2A, the SKA3 mRNA expression in A549 cells was reduced to 14.7% and 19.2% after transfection with si-SKA3- 1 and si- SKA3-2 for 48 h, respectively. The SKA3 protein expression levels were detected by western blot analysis and similar down-regulation of SKA3 was found (p<0.01, Figure 2B-C). Cells transfected with si-SKA3- 1 were used for subsequent experiments due to the higher knockdown efficiency. MTT experiments showed that the OD value of si- SKA3 group was visibly lower than that of si-con group at 24 h, 48 h and 72 h (p<0.01, Figure 3A). Colony formation assay revealed that knockdown of SKA3 notably reduced the number of formatted colonies (p<0.01, Figure 3B-C). In addition, wound healing assays were performed to investigate the migration ability of A549 cells. The results showed that cells with depleting SKA3 presented shorter migration distance (35.8%) compared to cells in si-con group (p<0.01, Figure 4A-B).

In addition, we transfected pcDNA3.1-SKA3 into A549 cells to overexpress SKA3. The effects of overexpression of SKA3 on A549 cell viability and motility were also detected. The results showed that overexpression of SKA3 significantly promoted cell proliferation (p<0.01, Figure 3E-F) and migration (p<0.01, Figure 4C-D). Collectively, these data suggested that SKA3 contributes to promoting lung adenocarcinoma cell growth and motility.

**Figure 5:**
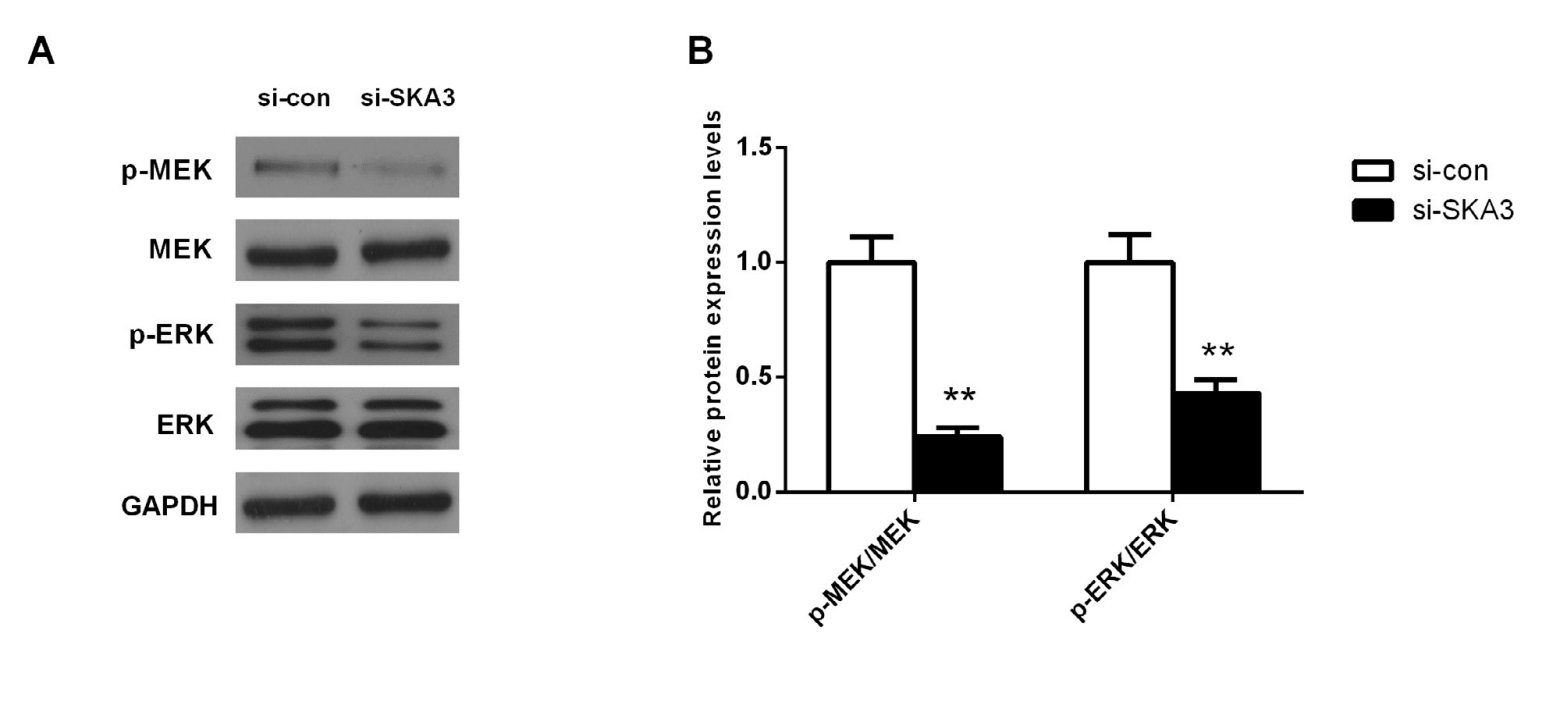
Down-regulating SKA3 suppressed the activation of MAPK signaling pathway. (A) The protein expression levels of p-MEK, MEK, p-ERK and ERK in A549 cells was determined by western blot. (B) The expression ratio of p-MEK/MEK, p-ERK/ERK. Columns, mean (n=6); bars, SD. **P<0.01, vs. expressions of these proteins in A549 cells in si-con group.

### 3.4 Down-regulation of SKA3 inhibits MAPK signaling pathway *in vitro*

The ERK/MAPK signaling pathway has been known to be involved in many cellular processes e.g. proliferation, migration and apoptosis [10]. Hence, western blot analysis was carried out to study the influences of silencing SKA3 on ERK/MAPK signaling pathway-related proteins expression. Compared to the si-con group, the protein expression ratios of p-MEK/MEK and p-ERK/ERK decreased significantly in the si- SKA3 group (Figure 4A-B, p<0.01). These data indicated that depleting SKA3 remarkably retards the activation of ERK/MAPK signaling pathway.

## 4 Discussion

Aberrant SKA3 expression has been observed in prostate cancer [11] and colorectal cancer [9, 12]. In the present study, we also observed the up-regulation of SKA3 in lung adenocarcinoma compared with that in normal lung tissues based on the mRNA expression data obtained from the public database. In addition, our results indicated that knockdown of SKA3 inhibited A549 cell growth and metastasis while over-expression of SKA3 promoted its growth and metastasis, possibly through inhibition of the MAPK signaling pathway.

The Ska protein complex is a heterotrimeric complex of Ska1, Ska2 and Ska3, which cumulates on spindle microtubules and at kinetochores following nuclear membrane disruption, becoming most abundant on kinetochores at metaphase [13]. Previous studies reported that SKA3 expression was enhanced during development from colorectal adenoma to colorectal cancer within individual patients [9]. Here, we initially studied the mRNA expression level of SKA3 in lung adenocarcinoma tissues based on the public database and determined that it is overexpressed in lung adenocarcinoma tissues compared to the normal lung tissues (Figure 1A-D). This overexpression is also observed in lung adenocarcinoma cells lines A549 and NCI-H460 (Figure 1E). Kaplan-Meier analysis showed that higher SKA3 expression is associated with lower overall survival rates (Figure 1F), indicating that SKA3 might be a prognosis predictor in lung adenocarcinoma patients. The clinical data indicated that SKA3 expression was obviously correlated with age (p=0.035), gender (p≤0.001), pathologic-stage (p≤0.001), pathologic-N (p≤0.001) and pathologic-M (p=0.046). These results strongly suggest that SKA3 might act as a promoter in lung adenocarcinoma development.

Cell proliferation requires the co-ordination of many proteins to accurately divide a cell into two daughter cells [14]. Previous studies revealed that knockdown of SKA3 by RNAi arrests mitosis in metaphase with an activation of the sister chromatid cohesion and sister chromatid separation [7, 13, 15]. In addition, depletion of SKA3 presents an inhibitory effect on cell growth and induces apoptosis in colorectal cancer cells [9]. Recently, Rong Hu et al. revealed that SKA3 exhibited a promoting effect on cell proliferation and migration in cervical cancer [16]. Consistent with their results, we found that the proliferation of A549 cells was substantially inhibited after knockdown of SKA3 (p<0.01, Figure 3A), indicating that SKA3 might function as a promoter in A549 cell proliferation. Collectively, these data indicated that overexpression of SKA3 is essential for cell division. Disturbed invasion and migration are two of the basic characteristic of tumors [17]. In HT29 and HCT116 cells, researchers found that knockdown of SKA3 remarkably reduced cell migration and invasion ability [9]. Through use of a wound healing assay, we also observed a substantial suppression of cell migration after depletion of SKA3 in our present study (p<0.01, Figure 3B). These results suggested that SKA3 might be used as a novel target for lung adenocarcinoma therapy.

It is well known that the MAPK signaling pathway is involved in many cell processes e.g. proliferation, division, migration and apoptosis [18]. Therefore, we determined the protein expression levels of MAPK signaling pathway-related proteins by western blot analysis. Our results showed that depletion of SKA3 makes the expression ratios of p-MEK/MEK and p-ERK/ERK reduce substantially (p<0.01, Figure 4). Therefore, we supposed that the MAPK signaling pathway may be involved in the inhibition of cell growth and migration caused by SKA3 depletion.

In conclusion, this study clearly demonstrated that the up-regulation of SKA3 expression is positively associated with lower disease-free survival in lung adenocarcinoma patients. Further validation and characterization of its role in larger human cohorts could help us have a better understanding of the underlying molecular mechanisms of lung adenocarcinoma.
